# Shoulder-wearable soft robot for assisting older adults in scapular stretching

**DOI:** 10.1017/wtc.2026.10044

**Published:** 2026-06-11

**Authors:** Kosuke Isobe, Masakazu Hirokawa, Yasuhiro Suzuki, Hideki Kadone, Yukiyo Shimizu, Kenji Suzuki

**Affiliations:** 1 Artificial Intelligence Laboratory, University of Tsukuba, Japan; 2 Data Science Laboratories, NEC Corporation, Japan; 3 Department of Rehabilitation Medicine, University of Tsukuba Institute of Medicine, Japan

**Keywords:** soft wearable robotics, rehabilitation robotics, mechatronics, design

## Abstract

We propose a wearable soft robot that assists with individualized scapula adduction and abduction for thoracic stretching in respiratory rehabilitation. Although thoracic stretching is known to be effective for respiratory rehabilitation, the range of motion of older adult patients narrows with age, and long-term external aid by physical therapists is required. The proposed robot consists of a soft and shoulder-wearable brace and cable-pulling mechanism to apply rotational torque on shoulders, resulting in stretching the thorax and scapulae. We designed the pulling mechanism by modeling the humeral head trajectory during stretching by a therapist and reproducing it with two linear actuators pulling the right and left shoulders simultaneously, based on position control aimed at achieving a target tension. The main results of validation experiments with older adults confirmed that the robot-assisted stretching was able to perform scapular stretching similar to that of a physical therapist.

## 1. Introduction

Chronic obstructive pulmonary disease (COPD) was the third leading cause of death worldwide in 2019 (Seattle, WA: Institute for Health Metrics and Evaluation, 2019). COPD is more common in older adults (Halbert et al., 2006). In addition, COPD, caused by irreversible and slowly progressive lung lesions, is a chronic condition (COPD Guideline 6th Edition Development Committee, 2022). It requires patients to undergo long-term treatment and support to prevent the gradual progression of the disease. Among respiratory rehabilitation, exercise therapy is one of the fundamental components of COPD treatment, which aims to improve physical activity by combining respiratory muscle stretching, lower-limb muscle training, and strength training of the upper limb and respiratory muscles. Respiratory muscle stretching is a technique for improving the mobility of respiratory muscles and respiratory accessory muscles, such as the diaphragm and intercostal muscles through upper-limb movements, including scapular movements that are effective for reducing dyspnea (COPD Guideline 6th Edition Development Committee, 2022) and increasing chest expansion (Rehman et al., 2020). The effectiveness of exercise therapy in maintaining physical function, such as a 6-min walking distance and the COPD assessment test, has been verified (Zanaboni et al., 2017). As the active range of motion (ROM) of the shoulder and scapula decreases with aging (Barnes et al., 2001; Endo et al., 2004), older adults may not be able to move their shoulders and scapula through a full ROM with active stretching alone. Consequently, for older adults who cannot achieve a full ROM, externally assisted stretching becomes crucial. Specifically, manual therapy techniques targeting the thoracic region have been shown to provide immediate improvements in respiratory function (e.g., FEV ${}_{1.0}$ ) and reduce dyspnea in patients with COPD (Yilmaz Yelvar et al., 2016).

Manual therapy by physical therapists is a technique to assist in expanding the ROM by applying external force. In manual therapy, the physical therapists mobilize the shoulder by grasping and moving it, thereby activating shoulder adductor muscles such as the pectoralis major, which secondarily enhances thoracic mobility. While patients themselves can stretch with external force using elastic bands and stretching machines, physical therapists have the advantage of being able to adjust the force, direction, and timing appropriately. Another advantage of manual therapy is that COPD patients often have musculoskeletal disorders, which may benefit from manual intervention (Chen et al., 2017). Thus, physical therapists are essential, and the demand for physical therapists is estimated to increase (Ministry of Health, Labour, and Welfare, 2019).

Especially in a super-aging society such as Japan, the burden on physical therapists is significant. Therefore, the ability to consistently provide such high-quality interventions is largely dependent on the experience and skills of physical therapists, and there are also issues such as physical burden, time constraints, and regional maldistribution. Furthermore, there is a need to realize individualized rehabilitation based on more objective and quantitative data and to provide continuous intervention support outside of facilities, such as at home. Therefore, there is a need for robots that can assist or supplement the work of physical therapists.

There are several challenges in reproducing thoracic stretching performed by physical therapists using robots. First, it is necessary to control not only the upper arm angle but also the humeral head position. Robotic systems capable of supporting these high degrees of freedom (DoF) are often bulky. Second, physical therapists qualitatively adjust the force application and movement direction for each individual based on the shoulder reaction force perceived in their hands during stretching. By perceiving shoulder reaction forces and the sensation at the final ROM, known as the end-feel (Cyriax, 1978), physical therapists provide optimal stretching tailored to individual patients. Such highly individualized adjustment is also a crucial factor in robotic interventions. However, the perception of end-feel can vary depending on the skill level of the physical therapist (Tasaka et al., 2019). Furthermore, although quantification based on force–displacement characteristics (Maitland and Kawchuk, 1997) has been proposed, this approach has been inadequate for complex joints such as the shoulder.

Therefore, in this study, we empirically aim for movement up to the final ROM by using the judgment of the patient, limitations imposed by hardware, and real-time measurement of force–displacement values. In addition, the size of the robot needs to be small enough to be used at home. Therefore, this study aims to reproduce the thoracic stretching intervention of a physical therapist using a wearable robot.

This work targets a passive therapeutic intervention – scapular stretching – specifically designed to support respiratory rehabilitation in older adults. Using a low-degree-of-freedom cable-driven system to manage a complex joint presents a highly compact alternative to traditional rigid rehabilitation exoskeletons. Our main contributions are as follows:

1. Proposed a shoulder-wearable soft robot for assisting scapular adduction and abduction stretching that adjusts the intensity according to the individual.
2. Clarified the mechanical properties of the proposed robot.
3. Verified the feasibility based on the scapular movement of younger and older adults.

## 2. Related works

There are several robotic methods to assist the wider ROM of people’s shoulder joints with different purposes and mechanisms. Several reviews have been published on upper-limb exoskeletons (Gull et al., 2020; Dhatrak et al., 2024). However, few exoskeletons or robots have been designed to assist older adults in respiratory muscle stretching.

### 2.1. Passive shoulder movement assistance

Stretching machines are used to improve muscle flexibility and strength. The Chest Spread (Park Corp.), a stretching machine, is designed to stretch chest and back muscles (Chest Spread, 2020). It consists of a simple mechanism that does not require the control of human joint angles. With the aid of this machine, the user can perform thoracic stretching without the therapist’s support. However, since this machine was designed to adjust its intensity based on the lower-limb movement (e.g., pedaling), its use is challenging for older adults with limited lower-limb mobility.

Passive mechanisms are used in industrial exosuits to assist shoulder motion (Tian et al., 2024). However, many mechanisms use gravity-compensation mechanisms to support long-duration tasks, making it difficult to apply them to stretching scapulae.

### 2.2. Robotic movement assistance

Upper-limb exoskeletons are used for medical and rehabilitation purposes such as neurorehabilitation, musculoskeletal rehabilitation, and motion assessment. For shoulder or scapular rehabilitation purposes, these exoskeletons provide high DoF control of the shoulder. Gopura et al. (2009) developed a 7-DoF upper-limb exoskeleton robot that can assist shoulder internal/external rotation. The robot is capable of supporting shoulder rotation through a mechanism that adjusts to the shoulder rotation center displacement. In addition, B Kim and Deshpande (2017) developed an exoskeleton system, “Harmony,” that can support glenohumeral joint and upper arm movements. The robot uses a 2-DoF mechanism to control the position of the humeral head to adjust to the displacement of the center of rotation of the shoulder. However, exoskeletons that support shoulder joint movements for bilateral configurations such as “Harmony” are often bulky due to their complex mechanisms to support movements with many DoFs (Rosen et al., 2014; Kim and Deshpande, 2017; Gull et al., 2020; Dhatrak et al., 2024).

Several exoskeletons or exosuits for assisting activities of daily living (ADL) for occupational purposes are more compact than exoskeletons for rehabilitating patients with paralysis (Jalal et al., 2024; Tian et al., 2024). For example, Kwok and Yu (2024) developed a lightweight bilateral underactuated upper-limb exoskeleton for bimanual ADL. In addition, Kim et al. (2018) developed a soft exosuit for industrial applications. However, these exoskeletons only provide assistance or gravity compensation for the shoulder abduction/adduction and shoulder extension/flexion and thus cannot achieve scapular adduction/abduction. They achieve compactness by treating humeral head displacement as an acceptable or negligible misalignment.

Continuous passive motion (CPM) machine is a mechanical device that supports a joint and can be set to move slowly through a designated ROM to promote controlled movement in the operated joint (Intervention applications, 2013). For example, the Kinex Shoulder CPM machine (Kinex Medical Company, 2018) is designed to move the patient’s shoulder joint through a prescribed ROM over a specified duration. However, most CPM machines do not consider changes in the position of the humeral head.

Some robots for respiratory rehabilitation assistance have also been studied (Zhu et al., 2015; Lee et al., 2022; Zhang et al., 2024; Zhou et al., 2025). Zhou et al. (2025) have proposed a bedside assistive robot using pneumatic actuators. This robot is a soft robot and is more comfortable than rigid robots. However, because it uses pneumatics, it requires the use of air compressors and other equipment, making it difficult to use at home.

### 2.3. Cable-driven wearable approach

Among the robotic assistance approaches, cable-driven wearable robots for the shoulder are suitable for home use due to their lightweight and compact design. Several cable-driven mechanisms are designed to support upper-limb movement, including the displacement of the humeral head (Samper-Escudero et al., 2020; Georgarakis et al., 2022). However, most methods are intended to assist with daily life activities and reduce the burden of work. Therefore, these cable-driven exoskeletons are designed to support the arm-raising motion based on the scapulohumeral rhythm; hence, they are not sufficient to assist the scapular adduction/abduction for thoracic stretching (Thalman and Artemiadis, 2020).

Several studies suggested wearable robots for resistance training that consider the movement of the humeral head. Park et al. developed a wearable fitness device for upper-limb exercise that constitutes cable-driven actuation to control the resistance profiles (Park et al., 2023). In addition, Pyeon et al. (2024) developed a cable-driven exosuit for upper-limb home fitness, capable of transmitting bidirectional resistance and applying focused loads to specific muscles utilizing cable routing. However, the primary objective of resistance training is strengthening muscle strength and muscular endurance, not to control the position or increase the ROM of the humeral head. Although many related studies have shown that the shoulder can assist with the ROM required for ADL, few studies have examined the effectiveness of robotic stretching for older adults in comparison with manual therapy.

We focus on a wearable robot with a cable-driven system to achieve stretching at home. A model is needed for movements involving scapular adduction/abduction. There is a musculoskeletal model for the scapula and shoulder, but it is difficult to simplify as much as the scapulohumeral rhythm, and the high DoF make the robot bulky. Therefore, the design is aimed only at supporting the movement of thoracic stretching.

A cable-driven robot for thoracic stretching has also been proposed by our group (Isobe et al., 2023). This device was designed to support the adduction and abduction of scapulae by controlling the position of the humeral head using a 2-DoFs cable-driven mechanism. However, in the study, due to safety concerns and to align the intervention conditions, the same control parameters (e.g., pulling force) were applied to all participants regardless of their physical size and/or strength. As a result, the robotic stretching was unable to provide sufficient support regarding the ROM compared to the manual stretching by a therapist. Therefore, the pulling force needs to be adjusted to the individual to ensure that the robot provides a sufficient ROM. However, that study was limited to younger participants, leaving its effectiveness for older adults unverified. Therefore, verification is also needed for older adults.

To address these limitations and ensure clinical applicability, the current study incorporates the following specific advancements compared to our previous work (Isobe et al., 2023):

- an individualized target tension procedure based on user feedback;
- a modified spine fixation mechanism to support the C7 reference point;
- a mechanical characterization of Bowden cable friction effects; and
- a feasibility study with older adults and comparison to a physical therapist.

### 2.4. Summary of the related works

Robots such as the upper-limb exoskeletons and exosuits in the previous studies mentioned above have not been investigated for the purpose of assisting thoracic stretching. To achieve thoracic stretching, controlling the humeral head position based on the understanding of the scapular motion is crucial. However, due to the high degree of freedom of the scapular movement, conventional exoskeleton approaches require a large mechanism. To make the system suitable for home use, it is essential to create a simpler mechanism. Exoskeletons and wearable robots designed for thoracic stretching lack performance verification of their effect on actual users’ range of movement, particularly for older adults.

Therefore, in this study, we propose a shoulder-wearable robot with a 2-DoF flexible cable-driven mechanism. We propose a control that varies the intensity of the stretching, which was fixed in our previous study (Isobe et al., 2023), according to the individual, and to characterize the mechanical properties of the proposed mechanism. We also verify the feasibility of the proposed robot for scapular adduction/abduction assistance through experiments with younger and older adults.

## 3. Methodology

### 3.1. Scapular adduction/abduction support robot

**Figure 1.**
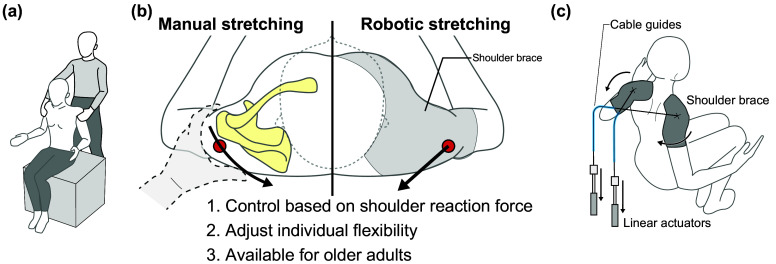
(a) A physical therapist performing thoracic stretching. (b) Overview of the similarities between manual stretching and the proposed wearable robot to assist in thoracic stretching. The physical therapist grasps the shoulder and controls it based on the shoulder’s reaction force, while the proposed robot controls the shoulder, which is stabilized by a brace, based on the pulling force. (c) Overview of the proposed robot. Figure 1. long description.

The overview of the proposed robot-assisted scapular stretching is shown in Figure 1. We propose a method to reproduce manual therapy shown in Figure 1(a) by using a wearable robot shown in Figure 1(c). Our method aims to reproduce manual therapy by modulating the pulling intensity based on shoulder reaction forces, as shown in Figure 1(b), providing robotic stretching for older adults. The proposed system dynamically supports scapular adduction by cable pulling while the elastic components of the shoulder brace passively facilitate the return motion to scapular abduction.

**Figure 2.**
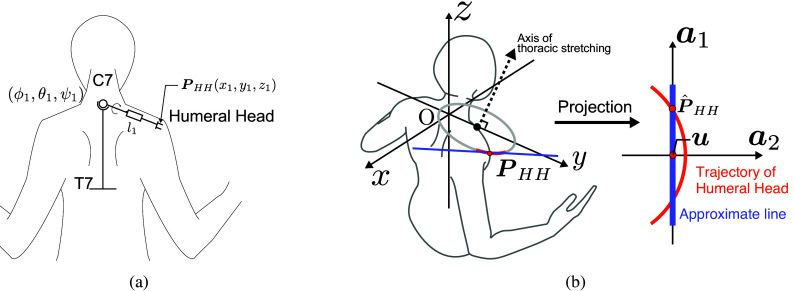
(a) Shoulder link model. (b) Trajectory of the right humeral head and approximate line. $P_{HH}$ : position of humeral head, ${\hat{P}}_{HH}$ : approximated position of humeral head. Figure 2. long description.

First, we measured the trajectory of the humeral head during manual therapy by a physical therapist. The following describes the approximation process for determining the pulling direction based on a model with C7 as the origin, as shown in Figure 2(a). The position of the humeral head $P_{HH}\left(x_{1},y_{1},z_{1}\right)$ in 3-dimensional space represented by C7 as the origin is expressed by the following equation:

(3.1) $$P_{HH}\;=\left(\begin{array}{c} x_{1}\\ y_{1}\\ z_{1} \end{array}\right)=\left(\begin{array}{c} l_{1}\sin\theta_{1}\cos\phi_{1}\\ l_{1}\sin\theta_{1}\sin\phi_{1}\\ l_{1}\cos\theta_{1} \end{array}\right)$$

${\hat{P}}_{HH}$ represents the approximated position of $P_{HH}$ on a linear trajectory consisting of the normalized first principal components as follows:

(3.2) $${\hat{P}}_{HH}=w_{1}a_{1}+u$$

where $a_{1}$ denotes the normalized first principal component, $w_{1}$ denotes the weight value obtained from the principal component analysis of $P_{HH}$ trajectory, and u denotes the center of gravity of the trajectory, as shown in Figure 2(b). Assuming that the change in $l_{1}$ and $l_{s}$ is proportional when the distance from C7 to $P_{HH}$ for another user is $l_{s}$ , the humeral head position $P_{HH}$ can be expressed by the following equation:

(3.3) $${\hat{P}}_{HH}=\frac{l_{s}}{l_{1}}\left(w_{1}a_{1}+u\right)$$

**Figure 3.**
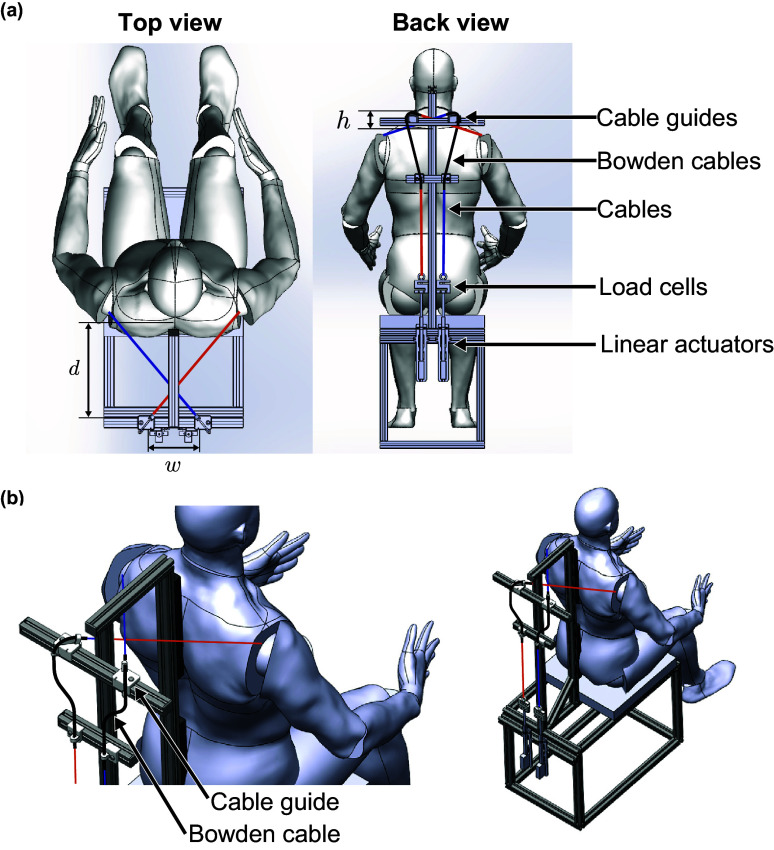
(a) Proposed robot configuration and cable guides positioning parameters. (b) The position of the cable guides. By adjusting the height and width of the cable guides, calculated by the shoulder width of the user, the pull direction of the cable-driven system can be aligned with the direction of movement of the user’s humeral head. Figure 3. long description.

The direction of this approximate line ( $a_{1}$ ) is assumed to be independent of the user’s body size. The pulling direction is fixed by adjusting the width and height of the cable guides according to the $l_{s}$ and $l_{1}$ . By controlling the cable pulling distance and tension with a cable-driven system using linear actuators, load cells, and Bowden cables, the 2-DoF robot can assist both scapular adduction/abduction simultaneously, as shown in Figure 3(a). When the distance from C7 to the cable guide is denoted as d , the distance between the cable guides, w , and the height from C7 to the cable guide, h , are expressed by the following equations:

(3.4) $$w=2\left|\frac{l_{s}}{l_{1}}u_{y}+|\frac{l_{s}}{l_{1}}u_{x}-d|\frac{a_{1y}}{a_{1x}}\right|,h=\frac{l_{s}}{l_{1}}u_{z}+|\frac{l_{s}}{l_{1}}u_{x}-d|\frac{a_{1z}}{a_{1x}}$$

An example of the cable guide mounting positions is shown in Figure 3(b). The width and height of the cable guides are adjusted according to the C7 position. Bowden cables are also used to reduce friction between the cable and the cable guide. By using redundant wiring, the Bowden cable adapts to changes in the position of the cable guide. A flexible shoulder brace is used for connecting the body and the cable-driven system.

It should be noted that the kinematic modeling of the humeral head described in this section is used only for hardware placement – specifically, to identify the optimal spatial arrangement of the cable guides in relation to individual anatomical landmarks. Since the current prototype cannot perform real-time shoulder position sensing, this model remains static and is not integrated into the active dynamic control loop.

### 3.2. Shoulder brace

The shoulder brace serves multiple functions beyond simply connecting the shoulder to the cable-driven system. The shoulder brace supports and stabilizes the shoulder joint, and the shoulder brace generally plays a role in pulling the shoulders toward the torso and preventing them from dropping downwards. Its function of restricting shoulder movement is used to achieve combined control around the shoulder joint. In addition, the torque required for shoulder motion varies with the angle of rotation of the upper arm (Kuechle et al., 1997). For example, Miyaguchi et al. (2008) attempted to support shoulder motion by CPM based on upper arm rotation measurements. The proposed robot uses a shoulder brace to prevent excessive upper arm inner/outer rotation change.

**Figure 4.**
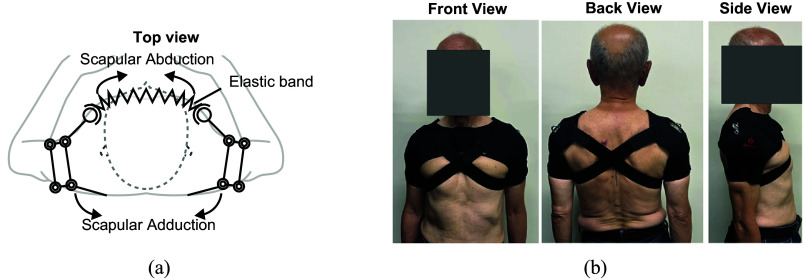
(a) Shoulder brace link model. The front elastic band passively supports scapular abduction. (b) An older person wearing a brace. Figure 4. long description.

Figure 4 shows a shoulder brace model and views of an older person wearing a brace. The shoulder brace is fixed with Velcro. Velcro is placed on the front of the body, making it easier for older adults to wear it alone. By pulling on the cable, the proposed robot can actively assist in scapular abduction, but releasing the cable does not actively assist in scapular abduction. Therefore, the braces on both sides are connected with elastic fabrics to passively support the movement of returning to the neutral posture during scapular abduction support.

### 3.3. Control

Review papers of the upper-limb control approach show that high-level interaction control strategies can be implemented in a range of methods, mainly including impedance/admittance-based strategies, adaptive control techniques, and physiological signal control (Rahman et al., 2010; Miao et al., 2018). The active motion of the scapula and the humeral head are modeled (FVD, 1994), but the passive joint ROM is unclear, so an intuitive and simple control method is required.

Approaching the final ROM poses ethical safety concerns; therefore, our control strategy aims to apply high-intensity stretching tailored to the individual. Freitas et al. found a greater ROM in joint stretching with high-intensity loading than with low-intensity stretching (Freitas et al., 2016). Therefore, we refer to the physical therapist’s direction of movement based on our previous study (Isobe et al., 2023) and aim for stretching with force set at a high intensity to suit the individual. As described above in Section 1, the greater the amount of pull on the shoulder, the greater the load required. The tendency of change in load and distance changes significantly near the final ROM. Therefore, in this study, to achieve high-intensity stretching, the ROM is set by setting the maximum tension in advance, rather than by detecting the end-feel and setting the controlled ROM. Depending on the individual’s physique and flexibility, the maximum tension varies and is determined based on the user’s diagnosis. During practical stretching by the robot, the maximum tension is set as the target tension, and traction of the shoulder and adduction of the scapula are realized by position-based force control. When holding the stretching state after the target tension is reached, the difference between the target tension and the current tension is used as an input for position control to cope with fluctuations in shoulder reaction force. Since the robot is not equipped with a system for sensing the user’s shoulder position, the humeral head position used in the model described in Section 3.1 is not used for control. We adopted this position-based force control strategy because setting safety thresholds based on 3D shoulder posture is technically difficult due to the joint’s complex 3D kinematics. Furthermore, electromyography (EMG) was not used because it is difficult to distinguish the stretching state from passive tissue resistance using muscle activity alone.

The robot operation is divided into two stages: the preparation and control processes. The preparation process aims to determine the individualized target tension $F_{\mathrm{ref}}$ based on user feedback. Following this, the control process executes a full stretching cycle consisting of three phases: loading, holding, and unloading.

The preparation process is an empirical protocol for determining the individualized target tension $F_{\mathrm{ref}}$ for each user. As explained in Section 1, the force increases as the pulling distance increases according to the force–displacement properties of a joint. Therefore, we estimate the maximum pulling force by gradually pulling the brace while the user wears the device. The force at a distance considered appropriate by the user is set as the target tension $F_{\mathrm{ref}}$ .

The robot’s control process strategy is mathematically described as follows. The control process performs stretching support based on the $F_{\mathrm{ref}}$ estimated in the preparation process. The robot uses a discrete-time, position-based force control method to ensure stable, safe stretching. The system updates the position command $x_{\mathrm{ref}}$ at each sampling interval based on the tension F[k] measured by load cells. At the start of the control process, the control logic for the next step k+1 is expressed as follows:

(3.5) $$x_{\mathrm{ref}}\left[k+1\right]=\left\{ \begin{array}{ll} x_{\lim} & F\left[k\right]<F_{\mathrm{ref}}\\ x\left[k\right] & F_{\mathrm{ref}}\leq F\left[k\right]<F_{\max}\\ x_{0} & F_{\max}\leq F\left[k\right] \end{array}\right.$$

where k is the discrete time step; $x_{\mathrm{ref}}\left[k+1\right]$ and x[k] are the next position command (mm) and current measured position (mm), respectively; F[k] and $F_{\mathrm{ref}}$ are the measured and target tensions (N), respectively; $F_{\max}$ is the safety limit tension (N); and $x_{\lim}$ and $x_{0}$ are the displacement limit and initial position (mm), respectively.

When the tension reaches the user-specific target $F_{\mathrm{ref}}$ , the system switches to the holding phase. At this transition, the position command $x_{\mathrm{ref}}$ is set to the current measured position x[k] to ensure a smooth transition to force maintenance. In this phase, the system uses a proportional control to maintain the force, where the position command is updated as follows:

(3.6) $$x_{\mathrm{ref}}\left[k+1\right]=\left\{ \begin{array}{ll} x\left[k\right]+K_{p}\left(F_{\mathrm{ref}}-F\left[k\right]\right)\Delta t & F\left[k\right]<F_{\max}\\ x_{0} & F_{\max}\leq F\left[k\right] \end{array}\right.$$

where $K_{p}$ is the proportional gain (mm/(N ⋅ s)). Finally, in the unloading phase, the position command is reset to $x_{0}$ . This allows the cable to loosen, enabling the scapulae to return to the neutral position, passively assisted by the elastic components of the shoulder brace. Implementation details are described in Section 4.2.

## 4. System overview

### 4.1. Mechanical design

**Figure 5.**
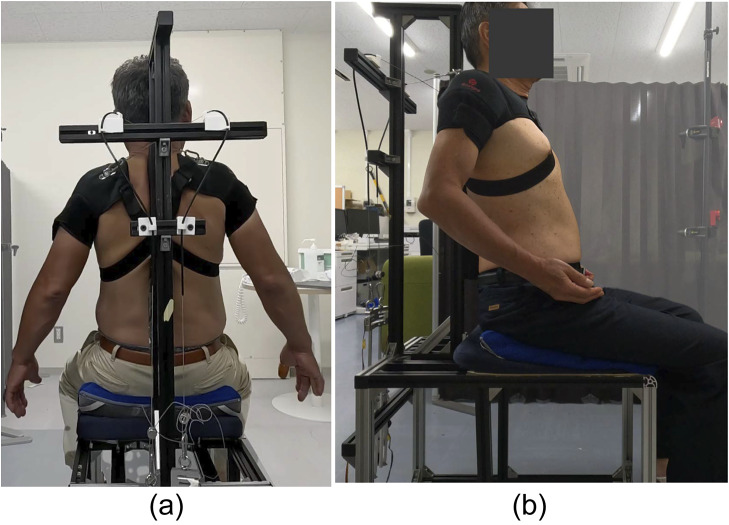
The developed robot: (a) Back view, (b) Side view.

Figure 5 shows the robot used in this experiment. The mechanism of this robot has several modifications from the previous robot (Isobe et al., 2023). First, the shape of the back cushion was changed to support the position of C7 (seventh cervical vertebra) since this robot models shoulder motion using C7 as a reference point. The upper thoracic spine is also fixed, as it is considered important to fix the spine to support thoracic spine joint mobilization.

Referring to the movements of physical therapists, they perform one dynamic stretch in about 2–3 s. For safety reasons, it is not necessary to control the actuator as fast as they do, but an actuator with a speed approaching that of a therapist’s movements is desirable. For force, the mass of the upper limb should be considered, and an actuator with a force greater than the traction force of the mass should be selected. The specifications of the selected actuators for the proposed robot are shown in Table 1.

**Table 1.** Specifications of the proposed robot Table 1. long description.

List	Value
Max speed (no load)	18 mm/s
Max force lifted (lowered)	90 N
Back drive force	200 N
Max static force	500 N

### 4.2. Robot behavior control

**Figure 6.**
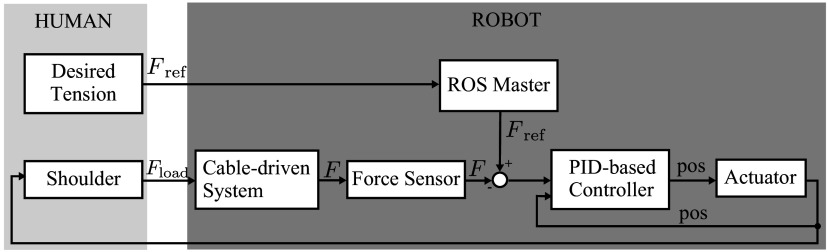
Block diagram of the proposed system.

According to Section 3.3, the robot realizes position-based force control. Figure 6 shows the block diagram of the proposed system. The linear actuators and motor drivers receive position control commands from a microcontroller called ESP32, which receives target value commands from ROS (Stanford Artificial Intelligence Laboratory et al., 2020). The target tension (represented by $F_{\mathrm{ref}}$ ) and repetition count are decided by the user. To enhance user intuition and ease of use, the target tension is set in units of kgf. The control frequency is about 40 Hz, and the position command $x_{\mathrm{ref}}$ is updated at a sampling interval Δt=0.025 s. This method allows the system to maintain the target tension even if the user moves slightly. For example, if a user suddenly changes posture or resists, causing F[k] to increase above $F_{\mathrm{ref}}$ , the error becomes negative. As a result, $x_{\mathrm{ref}}\left[k+1\right]$ becomes smaller than the current x[k] , and the cable tension is automatically released within 25 ms. If the tension reaches $F_{max}$ , the target is immediately reset to $x_{0}$ to loosen the cables and ensure user safety, and the robot terminates the stretching. In an emergency, the actuators can be stopped or extended to their maximum length by pressing the emergency stop button attached to the robot or the reset button on the operation control panel. In this study, safety is ensured by having the experimenter stay within 2 m of the robot when the user is using it and by immediately stopping the robot in case of an emergency.

In the loading phase of the control process, the target is set to $x_{\lim}$ at first. For safety, the robot is configured to reach $F_{\mathrm{ref}}$ in approximately 8 s, slower than the 2 s typical of manual therapy. To prevent prolonged strain if the target tension is not reached, a maximum duration of 12 s is set for this phase. Upon reaching $F_{\mathrm{ref}}$ , the system enters the holding phase and maintains the target tension for a maximum of 5 s.

In addition, the safety limit $F_{\max}$ is set to $F_{\mathrm{ref}}+19.6$ N. If F[k] exceeds $F_{\max}$ during any phase, the system immediately resets to $x_{0}$ and terminates the operation within 25 ms.

## 5. Performance evaluation

### 5.1. Evaluation of the proposed mechanism

**Figure 7.**
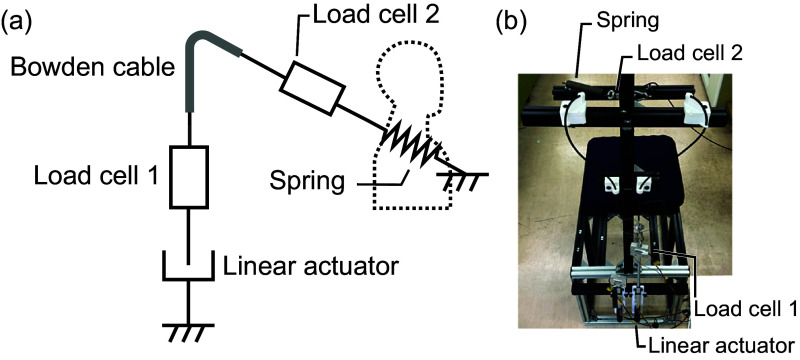
Experiment system in Section 5.1. (a) The mechanism of the system. (b) The appearance of the experimental system.

To evaluate mechanical properties and ensure safety, we evaluated the capability of the proposed cable-driven mechanism for scapular stretching. The objective was to verify that excessive tension is not applied when using the system on a human shoulder. Instead of using a human participant, we employed a stiff spring to approximate the force–displacement properties of the shoulder joint. This spring simulates the passive torque of a human shoulder, considering the relationship that tension increases proportionally with traction. The mechanism is shown in Figure 7. The model number of the spring used was AWY20-175 (MISUMI), with an initial tension of 12.75 N, a spring constant of .39 N/mm and maximum deflection of 167.5 mm. To evaluate the tracking performance of the end-effector during traction, a spring was selected that could accommodate the maximum displacement required for scapular adduction, simulating the compliance of the shoulder. However, determining an appropriate target tension is difficult due to limited consensus on the actual passive torque of the shoulder joint complex, particularly regarding data from scapular adduction movements used in this protocol. Therefore, to ensure safety in the user study, the maximum tension was set to 49.0 N (5.0 kgf) in this experiment, assuming users with a low maximum tolerable load. Traction started when the wire tension exceeded 1.2 N. The system was controlled to perform the stretching behavior as described in Section 3.3 for 10 times. The positions of the end-effectors of linear actuators were measured by potentiometers mounted on the actuators, and the tensions of the system were measured by the load cells. Only load cell 1 is used for control, and load cell 2 is only used to measure the force on the spring instead of the shoulder. Load cell 2 was not used for measurement in the user study because of the effect on measurement due to contact with the human body.

**Figure 8.**
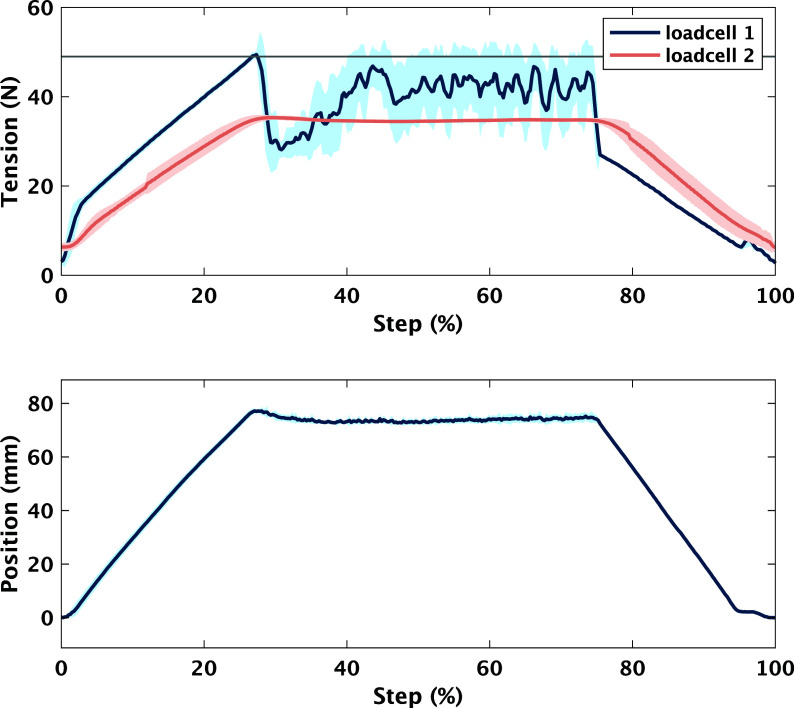
Changes in the mean and standard deviation of the tension of the two load cells and the pulling distance of the actuator over 10 trials. Figure 8. long description.

The mean values and standard deviation ranges for 10 trials are shown in Figure 8. The mean values are depicted with a bold line, with the standard deviation ranges shown as a shaded area. In addition, the target tension in the pulling and holding phase is depicted with a gray line. According to Figure 8, observation of the average tension in load cell 1 and the actuator’s pulling distance confirmed that traction ceased once the target of 49 N was reached. However, the tension on load cell 2 did not attain 49 N, and the average tension in load cell 2 reached a maximum of 35.3 N. Furthermore, it was observed that while load cell 1 exhibited oscillations after reaching the target tension, load cell 2 remained stable.

### 5.2. User study with younger adults

To evaluate the robot’s performance on younger adults, we measured the relative surface ROM of the scapula using motion capture while the participants wore the robot. Although surface-based measurements – such as the motion capture markers used in this experiment and the palpation used in the subsequent older adult study – are susceptible to skin motion artifacts and soft-tissue deformation, they provide practical indicators of the robot’s effect. Accordingly, the results presented in this section and Section 5.3 are framed as an assessment of relative surface range-of-motion rather than absolute skeletal kinematics.

#### 5.2.1. Participants

Eight male participants with no problems in the shoulder/scapula ROM and who did not habitually stretch their chest and back muscles using weights were recruited. The physical characteristics of the participants were as follows: age 21–26, height 173.1 ± 4.4 cm, and distance between right/left humeral heads 420–470 mm. The experiment was conducted with the approval of the Ethics Review Board of the Institution of Systems and Information Engineering at the University of Tsukuba (Approval No. 2022R709), and informed consent was obtained from each participant.

#### 5.2.2. Procedure

Before the main experiment, we determined the individualized target tension for each participant using the robot. To determine the individualized target tension, the experimenter manually pulled the cable to gradually increase the stretching intensity. The participants were given the following specific instruction: “We will gradually increase the stretching intensity. Please tell us when it reaches an intensity that feels ‘just right’ without pain. However, if you feel any pain during the adjustment or the experiment, please let us know immediately, and we will stop the device.” The tension value recorded at the point where the participant signaled was set as the target tension for the robotic intervention. This procedure was consistent for both younger and older adult groups. In this experiment, the target tension was set at over 49.0 N(5.0 kgf) to suit the individual. Note that, as indicated in the mechanical evaluation (section 5.1), the actual pulling force applied to the participant’s shoulder was lower than this set value due to friction loss in the power transmission.

Following this preparation, the experiment consisted of the following three stretching conditions:

- **Self-stretching:** Used to assess neutral posture. Participants received prior instructions on the method of active stretching movements.
- **Manual stretching:** Conducted by the experimenter (referred to as *Manual*).
- **Robotic stretching:** Conducted by the proposed robot (referred to as *Robot*).

Stretching was performed in the following order: Self-stretching → *Manual* → *Robot.* Although the experimenter was not a physical therapist, he received instruction from a physical therapist regarding the scapular stretching. A minimum rest period of 10 min was implemented between each condition to prevent residual effects and order effects.

**Figure 9.**
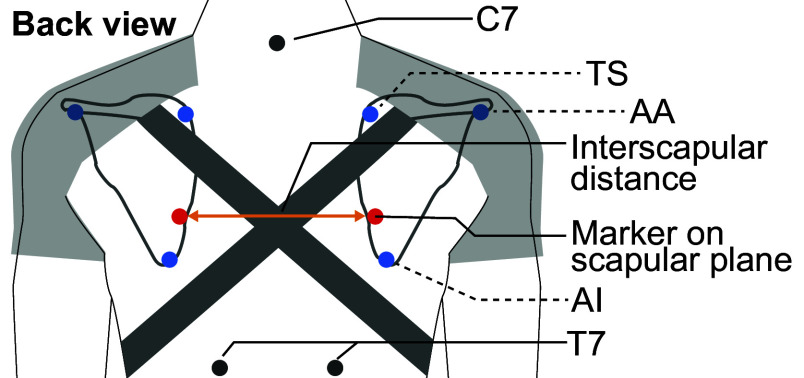
The position of retroreflective marker in the experiment of Section 5.2. Figure 9. long description.

To measure scapular motion, the positions of five markers attached to the back were measured using an optical motion capture system. The markers were attached at the positions of the ISB-recommended anatomical landmarks angulus acromialis (AA), trigonum spinae (TS), and angulus inferior (AI) (Wu et al, 2005), indicated as blue dots in Figure 9. However, in some cases, these markers were often occluded by the shoulder brace during the experiment. Therefore, referring to the fact that the distance between the AI and the spine is a common metric for evaluating scapular adduction, we attached markers at positions easily visible from the camera on a straight line connecting the AI and the TS, indicated as red dots in Figure 9. Then, the distance between these markers was calculated as the interscapular distance to evaluate scapular adduction. It should be noted that this metric relies on surface landmarks. Consequently, the measurements inevitably include the effects of soft tissue deformation and skin motion artifacts, which may differ slightly from the underlying skeletal kinematics. However, this method was considered sufficient for evaluating the relative changes in ROM within the scope of this study. Markers were attached to the skin over the scapula, ensuring the right and left markers were at the same height. Note that the position of the markers, the initial distance between the scapulae, and the position of the band of the brace differed among the participants due to the individual differences in shape and size of the scapula. Moreover, we attached markers in C7 and T7 as reference points to check for misalignment of the other markers.

**Figure 10.**
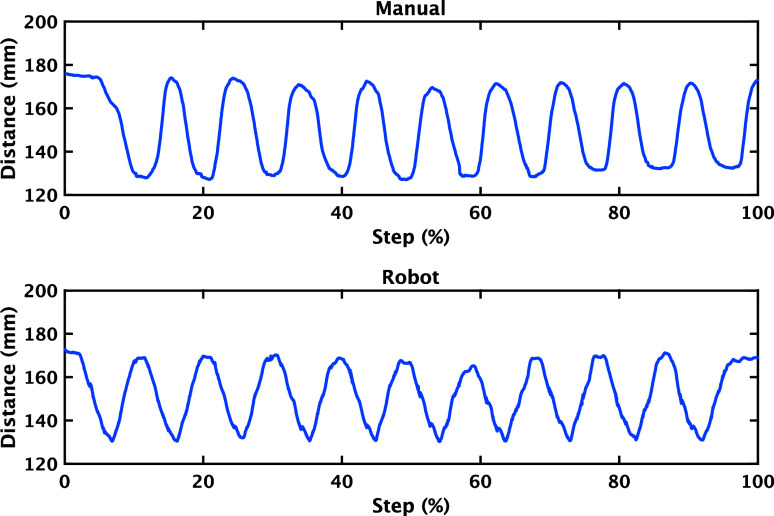
Interscapular distance of a participant in each condition.

An example of the change in interscapular distance for each stretching condition is shown in Figure 10. The minimum value of the interscapular distance in each condition was used to estimate the effect of stretching on scapular adduction.

#### 5.2.3. Statistical analysis

To identify the interscapular distance in the neutral posture (*Neutral*) as a reference, participants were asked to perform the stretching by themselves, and the maximum value of the interscapular distance during self-stretching was used. Pairwise comparisons between conditions were conducted using the Wilcoxon signed-rank test to assess statistical differences. To adjust for multiple comparisons, the Bonferroni correction was applied.

#### 5.2.4. Result

**Figure 11.**
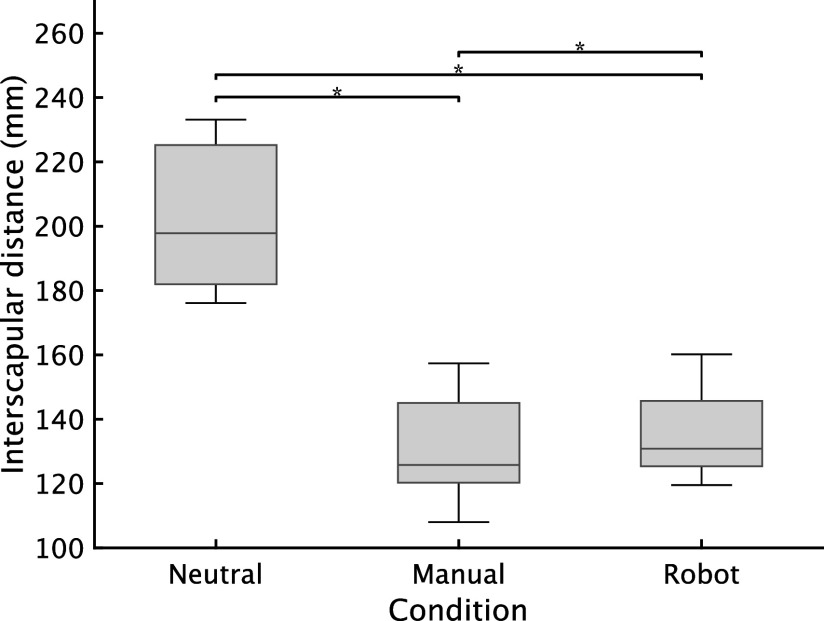
Box plot of the interscapular distance of participants in each condition, along with the results of statistical analysis *: p<.05 ).

The box plot of the interscapular distances for all participants in the *Neutral*, *Manual*, and *Robot* conditions is shown in Figure 11. Significant differences were confirmed between manual stretching and neutral posture and between robotic stretching and neutral posture ( W=0 , adjusted p<0.05 ). Wilcoxon signed-rank test showed a significant difference between robotic stretching and manual stretching ( W=0 , adjusted p=0.023<0.05 ). However, the mean difference between the two conditions was 5.1 mm (130.9 mm for the robotic stretching and 125.8 mm for the manual stretching). This difference was only 7.1% of the mean difference observed between neutral posture and manual stretching. In addition, no one complained of pain or discomfort during the stretching.

### 5.3. User study with older adults

To evaluate the robot’s performance and short-term effect on older adults, scapula–spine distances and respiratory function were measured with the robot worn by the older adults. The purpose of this experiment was to clarify the feasibility of robotic stretching for older adults compared to manual stretching by the physical therapist and its effect on the immediate improvement of respiratory function. The target tension was set at over 49.0 N (5 kgf) to suit the individual.

#### 5.3.1. Participants

The participants were nine males over 65 years, defined as early-old by WHO, with no diagnosis of shoulder problems. The participants had a mean age of 70.9 years (SD = 2.6, range: 68–74). The mean height of the participants was 168.7 cm (SD = 7.2, range: 158.0–176.0 cm), the mean weight was 70.4 kg (SD = 9.5, range: 62.5–86.0 kg), and the mean of body mass index (BMI) was 24.7 kg/ $m^{2}$ (SD = 2.5). This study was conducted with the approval of the Ethics Review Board of the Institution of Systems and Information Engineering at the University of Tsukuba (Approval No. 2022R709–4), and informed consent was obtained from each participant.

#### 5.3.2. Procedure

The procedure for determining the individualized target tension was consistent with that used in the younger adults study (Section 5.2). Based on the participants’ feedback regarding the maximum comfortable intensity, the target tension settings for the older adults ranged from 98.0 N (10.0 kgf) to 137.3 N (14.0 kgf) at the actuator side. Note that, as indicated in the mechanical evaluation (Section 5.1), the actual pulling force applied to the participant’s shoulder was lower than these set values due to friction loss in the power transmission.

**Figure 12.**
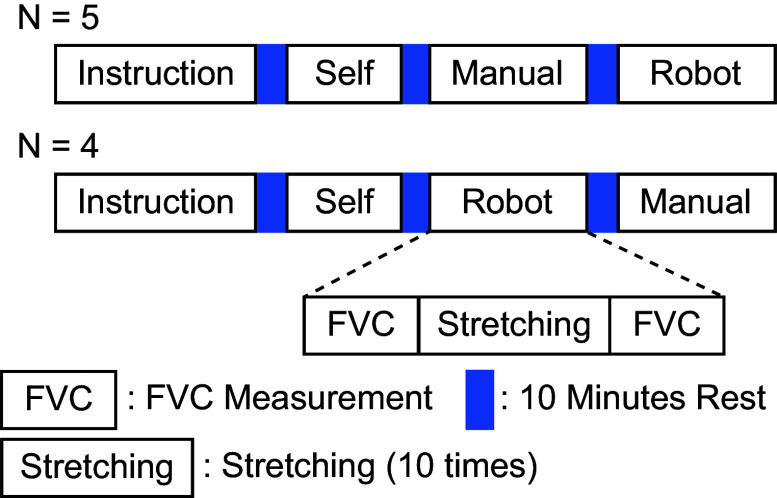
Measurement procedure in experiment of Section 5.3. Figure 12. long description.

Four conditions of scapula–spine distance were measured. (1) neutral posture (*Neutral*), (2) self-stretching, (3) manual stretching by a physical therapist (*Manual*), and (4) robotic stretching (*Robot*). Considering that the ROM of the scapula is generally increased by the physical therapist’s treatment or robotic stretching, self-stretching was measured at first. Self-stretching was performed to confirm the participant’s ROM and to confirm that it was safe to perform the robotic stretching. The procedure of this experiment is shown in Figure 12. The distances of *Neutral* conditions were measured before self-stretching. Five participants performed the robotic stretching first, followed by manual stretching, and the remaining four participants received manual stretching and then received robotic stretching. Participants performed stretching 10 times for each condition. In addition, the participants in the experiment measured FVC (forced vital capacity), FEV1.0 (Forced Expiratory Volume in 1 s), and the FEV1.0% (FEV1/FVC) using a spirometer (CHESTGRAPH HI-301 U, CHEST M.I., Inc.) before and after the two conditions (*Manual* and *Robot*) mentioned above.

**Figure 13.**
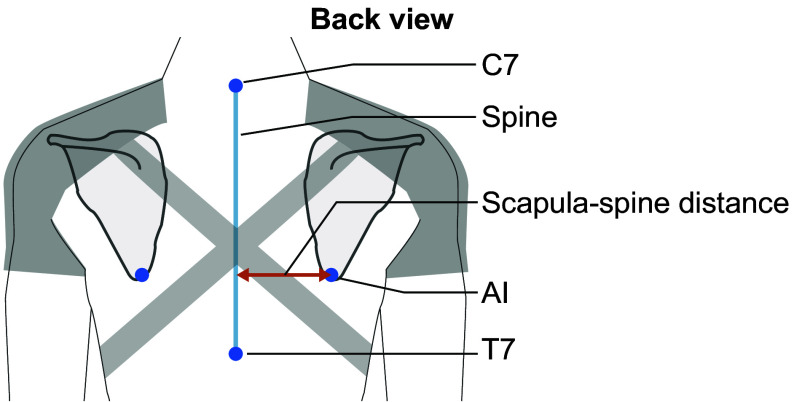
Measurement position in experiment of Section 5.3.

Figure 13 shows the scapula–spine distance used as an assessment parameter. We used an optical motion capture system in Section 5.2, but in this experiment, we used a digital caliper because the marker occlusion by the user’s and therapist’s bodies and waiting bare-chested is a burden for older adults. Although this metric provides a one-dimensional measurement of three-dimensional scapular motion, measuring the scapula–spine distance via palpation is a standard clinical practice in manual therapy for assessing scapular adduction (Johnson, 2012). Therefore, we considered this method valid and sufficient for verifying the feasibility of the robot-assisted stretching in this study. Using a skin marker pen, draw a line at the location of the spine and a dot at the location of the AI. Since the dots were written on the surface of the skin, the position of the dots (AI) can be displaced from the actual position of the scapula during stretching. To ensure consistency and accuracy, all measurements were performed by a single experimenter who had been trained in palpation techniques by a licensed physical therapist. The stretching was performed 10 times. The first five trials were excluded to allow for the convergence of initial shoulder brace displacement and to enable the experimenter to confirm stable robot operation. Consequently, the latter five times were measured. To ensure a steady state during the measurement in manual stretching, the physical therapist was instructed to hold the scapulae in the maximally adducted position for 5 s. The average value was calculated as the scapula–spine distance.

For respiratory function evaluation, FEV1.0 and FEV1.0%, which are commonly used for COPD assessment, were adopted for FVC measurement. Although there is a study showing significant differences in FVC and FEV1.0 values even in immediate manual therapy for COPD patients (Yilmaz Yelvar et al., 2016), it should be noted that this study was conducted on healthy older adults. The participants were instructed by a physical therapist on how to use the stretching device, practiced using it, and then rested enough before the measurement.

#### 5.3.3. Statistical analysis

To compare the scapula–spine distance across each condition, pairwise comparisons between conditions were conducted using the Wilcoxon signed-rank test to assess statistical differences. To adjust for multiple comparisons, the Bonferroni correction was applied. Adjusted p-values less than .05 were considered statistically significant.

The following statistical analyses were performed to compare the effects of stretching on respiratory function. To verify that there was no change in respiratory function before stretching, a Friedman test was performed on the respiratory function index before stretching. A Wilcoxon signed-rank test was then performed for each condition to verify changes in respiratory function before and after stretching.

#### 5.3.4. Result

**Figure 14.**
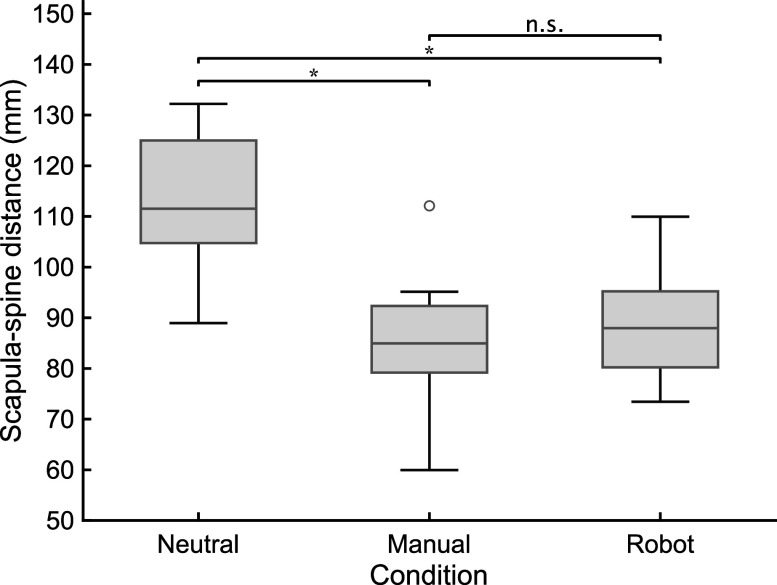
Box plot of the scapula–spine distance of participants in each condition, along with the results of statistical analysis in Section 5.3 (*:adjusted p<.05 ; n.s.: not significant).

Figure 14 summarizes the average scapula–spine distance of all participants for *Neutral*, *Manual*, and *Robot* conditions as box plots. There were significant differences between the condition of neutral posture and the condition of robotic stretching ( W=0 , adjusted p<.05 ) and between the condition of the neutral posture and the condition of manual stretching ( W=0 , adjusted p<.05 ). There was no significant difference between the manual stretching and the robotic stretching. In addition, the physical therapist interviewed the patients, but no one complained of pain or discomfort from stretching.

**Figure 15.**
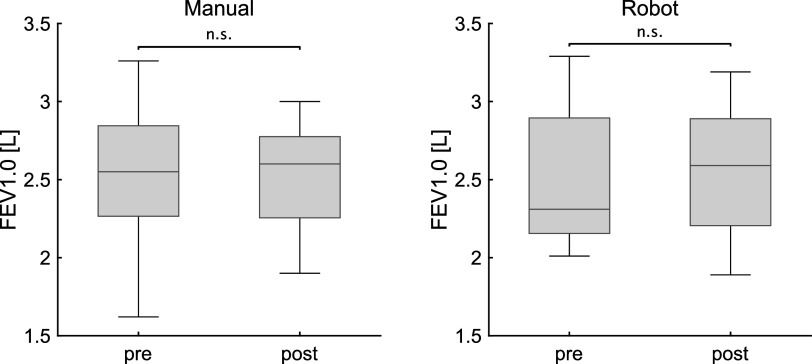
Box plot of pre-and post-FEV1.0 for each condition in Section 5.3 (n.s.: not significant).

The results of the pre-and post-FEV1.0 changes for each condition are shown in the box plots in Figure 15. Due to missing data, we analyzed data from eight participants for the *Robot* condition and nine for the *Manual* condition. Due to a technical error in the data logging process, data for one participant in the *Robot* condition were lost. Consequently, we analyzed data from eight participants for the *Robot* condition and nine for the *Manual* condition. It is noted that this data loss occurred randomly and was unrelated to the participant’s performance or fatigue. Friedman tests were conducted for the precondition in all stretching conditions. No significant differences were found when the significance level was set at 5%. Wilcoxon signed-rank sum tests were conducted on the FEV, FEV1.0, and FEV1.0% values pre- and post-stretching. No differences were found in all conditions when the significance level was set at 5%.

## 6. Discussion

### 6.1. Mechanical characteristics of the proposed mechanism

The mechanical evaluation detailed in Section 5.1, which utilized a spring to simulate the passive properties of the shoulder joint, provides a quantitative basis for interpreting the operational characteristics of the proposed cable-driven mechanism. During this experiment involving the mechanism of the proposed robot, the position and tension at the linear actuator and tension on the spring were monitored both until the target tension was achieved and after reaching that target. Understanding this behavior is crucial for validating the system’s performance and ensuring its safe application in user studies. We consider the responsiveness, stability, and safety of the proposed mechanism based on the behavior of the system.

#### 6.1.1. Responsiveness considerations

The tension response measured by load cell 2, as depicted in Figure 8 (Changes in the mean and standard deviation of the tension of the load cells and the pulling distance of the actuator), confirmed that the actual force exerted on the spring did not reach 49.0 N. A discrepancy was observed between the tension at the actuator and the tension at the end-effector. This phenomenon is interpreted as an inherent characteristic of the Bowden cable transmission, where internal friction causes inevitable force attenuation and hysteresis, as widely reported in cable-driven mechanisms (Chen et al., 2014). This finding clarifies that the force applied to the user is a fraction of the force measured at the actuator, a critical factor in understanding the system’s responsiveness.

#### 6.1.2. Stability considerations

Regarding stability, while oscillations were noted in the actuator-side tension (load cell 1) after reaching the target tension, the tension at the end-effector (load cell 2) remained stable. This suggests that the observed oscillations are a result of friction phenomena within the cable sheath and do not translate to unstable force delivery to the spring or user. Therefore, we interpret the system’s output as stable and well-damped, capable of maintaining a steady stretching force as intended.

#### 6.1.3. Safety considerations

Acknowledging the challenge of algorithmically detecting the clinical “end-feel,” our primary safety strategy relies on an empirical tension-setting protocol. This method uses direct user feedback to define a maximum force tailored to the individual’s tolerance, thereby preventing the robot from stretching beyond a safe and comfortable limit.

A key finding from the mechanical evaluation is the absence of tension overshoot at the end-effector (load cell 2). Despite the complexities of the cable transmission, the control system robustly prevented the applied force from exceeding the target tension. This behavior is critical, as it confirms the system will not apply sudden, excessive forces, making the empirically set tension limit a reliable and safe operational ceiling.

Finally, this combination of an empirical protocol and inherent system stability is supplemented by multiple physical fail-safes. These include an emergency stop button, a stop function accessible from the control GUI, constant experimenter monitoring, and a procedure for cutting the wires in an emergency, ensuring comprehensive safety throughout the user study. While the current protocol relies on user feedback, we acknowledge the potential for altered or delayed pain perception in older adults. To compensate for this vulnerability during data collection, conservative $F_{\mathrm{ref}}$ settings were adopted, and both an experimenter and a physical therapist continuously monitored participants for non-verbal signs of discomfort throughout the user study described in Section 5.3.

### 6.2. User study

According to Section 5.2, for younger adults, both stretching conditions were significantly different from neutral posture, confirming that both stretching conditions achieved scapular movement. Although a significant difference was observed between manual stretching and robotic stretching, the median difference was only 5.1 mm. The median difference between *Neutral* and *Manual* was 72.0 mm, indicating that the difference between *Manual* and *Robot* was relatively small at 7.1% of the difference between *Neutral* and *Manual.* Therefore, the proposed robot can provide a ROM support comparable to or slightly less than that of manual stretching for younger adults. Since adverse physical effects on the skin and reports of pain were not identified in the younger adults, it was determined that the experiment could be performed on the older adults.

According to Section 5.3, for older adults, there was a significant difference in scapula–spine distance for robotic stretching in comparison to the neutral posture as well as to manual stretching. In addition, no significant difference in scapula–spine distance was noted between manual and robotic stretching, indicating that the proposed robot can adequately support a ROM equivalent to that achieved through manual stretching.

This experiment on the participants of younger and older adults did not show that robotic stretching was significantly superior to manual stretching. In shoulder stretching, the shoulder’s ROM is determined by soft tissue stretching. Therefore, it should be noted that the ROM depends on the intensity of the passive stretching. In the user study with younger adults, a significant difference was observed between manual stretching and robotic stretching. This difference is likely attributable to the greater ROM and muscle mass in younger individuals, requiring forces that exceed the current system’s intended operational range for an older population. This outcome reinforces the appropriateness of the robot’s design parameters. The primary goal – providing therapist-equivalent support to older adults – was successfully achieved.

Furthermore, according to Section 5.3, no immediate improvement was observed in respiratory function tests. This lack of improvement is not surprising, given that the exercises in this experiment were only partially focused on respiratory muscle stretching, and general respiratory muscle stretching has minimal immediate effects on breathing. However, no adverse effects of robotic stretching on breathing were identified. Long-term experiments are required to assess the long-term effects of robotic stretching on respiratory function.

### 6.3. Positioning in soft wearable robotics

In recent years, research on soft wearable robots has advanced rapidly, leading to a wide range of applications. As indicated in a review by Thalman and Artemiadis (2020), the objectives of soft wearable robots have predominantly focused on the recovery and assistance of ADLs and the augmentation of strength, endurance, and motor capabilities, thereby centering on the reconstruction of active movements. However, while expanding the ROM is also mentioned as a potential objective, its application to the shoulder has often been overlooked. This study pioneers new potential applications for soft wearable robots by addressing this specific and underserved clinical need.

Among soft wearable robots, the cable-driven (or tendon-driven) method is a mainstream approach. In Section 2.4, we discussed several cable-driven soft wearable robots designed to assist the shoulder. This method is not limited to the shoulder; its principle of mimicking the human musculoskeletal system’s tendon-driven mechanisms is a common strategy for actuating various body parts. In the Exo-Glove Poly II (Kang et al., 2019), for instance, a tendon-driven mechanism is used to control multiple interconnected joints, such as those in the fingers, with fewer actuators. For lower-limb assistance, Harvard’s soft exosuit uses cable-driven actuation integrated with fabrics to control the hip and ankle, enabling it to provide support while accommodating joint alignment errors (Lee et al., 2018). Furthermore, the spine-inspired continuum soft exoskeleton (Yang et al., 2019), which also operates via cable-driven actuation, offers greater freedom of motion (flexion, lateral bending, rotation) than rigid exoskeleton designs. In our study, we also adopted a cable-driven approach, enabling us to propose a more compact system than rigid counterparts. Our unique contribution is demonstrating low-DoF control over a complex, high-DoF joint like the shoulder, where the cable-driven mechanism inherently accommodates misalignments while providing effective assistance.

Furthermore, according to a review by Bardi et al. (2022) on upper limb soft wearable robots, soft wearable robots’ control is often based on intention detection, IMU-based trajectory tracking, or EMG-based control. These control strategies are effective when the user is actively participating or when the operational ROM is predetermined. In our study, however, the user passively receives the stretching, and the target ROM is not the normal ROM but varies depending on the individual and their condition. Therefore, the novelty of our approach lies in the integration of force-based control alongside position-based control. This study can also be considered unique in that it aims for therapeutic “intervention,” in contrast to the many soft wearable robot studies that target synergy with the user’s active movements.

While applications for upper limbs and lower limbs have been reported previously, this study demonstrates that limited actuators can effectively address joints with high DoF, such as the shoulder joint examined here. This finding suggests the potential of soft wearable robotics for safe and adaptable rehabilitation applications with high human affinity.

### 6.4. Future directions

The findings of this study suggest several key directions for future research aimed at enhancing the robot’s utility and validating its clinical potential.

While the current empirical approach based on user feedback proved effective and safe, future work could explore more objective methods. For this purpose, one promising approach is to algorithmically detect the end-feel and the shoulder’s ROM by analyzing the force–displacement profile of the shoulder. While research on torque and end-feel detection for shoulder girdle mobilization has been insufficient, this experiment largely clarified user force profiles. Consequently, we aim to develop an automatic stop mechanism and verify its effectiveness in future work. To ensure more safety, control strategies that take into account the feedback of joint posture using IMU and friction of the cable transmission are needed, referring to previous studies (Georgarakis et al., 2020; Zhou et al., 2025; Zhu et al., 2025). Moreover, for respiratory rehabilitation, it is crucial to adjust not only the intensity of the movement but also the timing relative to breathing. Furthermore, although this study confirmed no user discomfort, future studies should incorporate quantitative assessments of user comfort and pain, such as the Visual Analogue Scale (VAS) and customized questionnaires. For example, a comprehensive tool like the recently developed “USE_WR 1.0” could be used (Park et al., 2025). This will enable a more thorough evaluation of the robot’s user-friendliness, especially when used with the target clinical population of older adults.

The primary objective of the proposed robot is to prevent a decrease in the ROM of the respiratory-related muscles around the thorax through robotic stretching, thus avoiding a decrease in respiratory function. While this initial study did not show immediate changes in respiratory metrics, it critically establishes the safety and feasibility required to proceed to the next phase of research. Numerous prior studies on respiratory muscle stretching have involved long-term experiments on respiratory rehabilitation, and it is imperative to replicate such studies. For instance, research by Yamada et al. (1996) on respiratory muscle stretching gymnastics (RMSG) observed significant differences in dyspnea. Furthermore, a study by Minoguchi et al. (2002) comparing inspiratory muscle training and RMSG found significant differences in maximum chest wall expansion and 6-min walking distance. Therefore, long-term experimentation and measurement of chest wall expansion and dyspnea using the VAS will be necessary.

## 7. Limitation

While the results of this study are sufficient to provide thoracic stretching, this study has several limitations that should be addressed.

First, the participants were healthy early-old adults (65–74 years) rather than COPD patients. This limits the validation of the system’s therapeutic effectiveness for its intended clinical application: older adults with COPD.

Second, our evaluation was confined to the immediate, acute effects of a single stretching session. The primary goal of manual stretching is to improve respiratory muscle flexibility and reduce dyspnea. Therefore, the present results cannot be used to infer long-term treatment effects, and a study design that is longer-term and examines multiple metrics is required.

Third, the feasibility of home use has not been fully verified since no home experiments were conducted in this study. The proposed system uses only a wearable robot for sensing and control and is as small as a chair, so it can be used anywhere, including in the home, as long as there is a power source. However, we believe that verification of ease of use and system stability, which are issues when an older person uses the system alone, is also necessary.

Fourth, the system has specific technical and modeling limitations. The shoulder model, based on a single healthy male participant, may not adequately represent anatomical variations. In addition, the system used the spring to simulate the mechanical behavior of the shoulder joint; however, the actual passive torque variation at the shoulder displays nonlinear characteristics and hysteresis effects. Moreover, the system’s force application is constrained by the actuator’s maximum output (90 N) and, more significantly, by frictional losses inherent in the Bowden cable transmission. This characteristic, quantified in the mechanical evaluation, likely contributed to the reduced effect in younger participants with higher joint stiffness. This technical constraint limits the versatility of the current prototype for populations requiring higher forces.

Fifth, the evaluation methodology has its own constraints. The use of one-dimensional distance metrics (interscapular and scapula–spine distance) provides a valid but incomplete picture of a complex, three-dimensional scapular motion. Furthermore, while no discomfort was reported, user comfort was not assessed with quantitative scales such as VAS for pain or a custom comfort questionnaire. While the inclusion of quantitative scales of comfort would have substantially strengthened our claims, it is also noteworthy that a recent study (Mohammed El Husaini et al., 2023) has pointed out challenges in the repeatability and consistency of VAS for assessing exoskeleton comfort, suggesting that careful methodological design is required for its application. In addition, although a 10-min rest period was implemented between conditions to mitigate carry-over effects on respiratory function, this may not have completely eliminated the influence of previous stretching sessions. A randomized controlled trial would be necessary to address this methodological limitation.

Finally, we acknowledge a limitation regarding the experimental order in the younger adult study, which was fixed (Self-stretching → Manual stretching → Robotic stretching) rather than randomized. This specific order was necessary due to setup constraints: removing the tight-fitting wearable robot posed a high risk of displacing the reflective markers attached directly to the skin, which would have compromised the consistency of the motion capture data for subsequent conditions. To mitigate potential order effects such as fatigue or tissue preconditioning, sufficient rest intervals were provided between trials; however, the influence of the fixed order cannot be completely ruled out. Although a fixed order may introduce bias, we assume that such order effects (e.g., learning, fatigue, or viscoelastic tissue changes) were minimal, considering the short duration of the intervention and the healthy young demographic of the participants.

## 8. Conclusion

In this study, we proposed a soft wearable robot that is adjusted based on the strength of individuals and conducted experiments with younger and older adults. The results showed that the proposed robot provides a sufficient ROM for both younger and older adults, with no significant differences observed between robotic and manual stretching in older adults. This suggests the potential for alleviating physical therapists’ workload while maintaining therapeutic quality. Future work will focus on long-term studies to evaluate chronic effects on respiratory function and ROM, particularly in clinical populations.

## Data Availability

All necessary data are included in the manuscript.

## Figure 1. Long description

Panel one at the left depicts a therapist seated behind a patient, holding the patient’s shoulder for thoracic stretching. Panel two in the center is divided vertically; the left side is labeled Manual stretching and shows a hand grasping the shoulder, with arrows indicating control based on shoulder reaction force. The right side is labeled Robotic stretching and shows a shoulder brace, with arrows indicating control based on pulling force. Three numbered points below list: 1. Control based on shoulder reaction force, 2. Adjust individual flexibility, 3. Available for older adults. Panel three at the right illustrates the proposed robot, showing a person wearing a shoulder brace, cable guides, and linear actuators connected to the brace, with arrows indicating movement direction.

Navigate back to Figure 1..

## Figure 2. Long description

Panel a on the left shows a simplified human torso with arms extended, highlighting the right shoulder. The C7 and T7 vertebrae are labeled as spatial anchors. The humeral head is marked with a circle, and its position is denoted as P sub H H with coordinates x sub 1, y sub 1, z sub 1. The shoulder link l sub 1 connects C7 to the humeral head. Three rotation angles phi sub 1, theta sub 1, psi sub 1 are indicated at the shoulder joint. Panel b, to the right, overlays a three-dimensional coordinate system with axes x, y, z centered at O. The axis of thoracic stretching is shown as a dashed line. The position P sub H H is marked by a red dot, and a blue line connects O to P sub H H. An arrow labeled ‘Projection’ points rightward to a two-dimensional plot with axes a sub 1 and a sub 2. On this plot, the trajectory of the humeral head is shown as a curved orange line labeled ‘Trajectory of Humeral Head,’ while a blue line labeled ‘Approximate line’ runs close to it. The projected position, P hat sub H H, is marked by a red dot on the trajectory. The variable u is shown along the trajectory curve.

Navigate back to Figure 2..

## Figure 3. Long description

The diagram contains two main sections. In the top row, the left panel shows a top view of a seated human model within a rectangular robot frame, with two colored cables (blue and red) extending from the frame corners toward the shoulders. The width (w) and depth (d) of the frame are labeled. The right panel presents a back view, labeling cable guides at shoulder height (h), Bowden cables, cables, load cells, and linear actuators along the spine and frame. Arrows indicate the cable routing from the actuators upward through the guides. In the bottom row, the left panel is a close-up perspective of the cable guide and Bowden cable positioned near the user’s shoulder, with labels pointing to each component. The right panel shows a wider perspective of the user seated in the robot, highlighting the cable path and guide placement relative to the shoulder. The spatial relationship between cable guides and user anatomy is emphasized, with cable direction aligned to the humeral head movement.

Navigate back to Figure 3..

## Figure 4. Long description

On the left, a line diagram shows a top view of a shoulder brace. At the center is a dashed circle representing the torso. Outward from the center, two arms extend, each with jointed segments. At the top, a zigzag line labeled elastic band connects the two arms. Arrows labeled scapular abduction point outward from the elastic band, while arrows labeled scapular adduction point inward along the arms. On the right, three photos display an older person wearing the brace. The first photo shows the front view, with the brace crossing the chest and shoulders. The second photo shows the back view, where the brace straps cross in an X shape over the upper back. The third photo shows the side view, highlighting the brace’s fit around the shoulder and upper arm.

Navigate back to Figure 4..

## Figure 8. Long description

The top panel displays tension in newtons on the y-axis versus step percentage on the x-axis. Loadcell 1 shows a sharp rise to about 45 newtons at 20 percent, then fluctuates between 40 and 50 newtons until 80 percent, followed by a steep decline. Loadcell 2 rises more gradually, peaking near 35 newtons at 20 percent, then plateaus around 30 newtons until 80 percent, before decreasing. Shaded regions indicate standard deviation over 10 trials. The legend at the top right identifies loadcell 1 in blue and loadcell 2 in red. The bottom panel shows actuator position in millimeters on the y-axis versus step percentage on the x-axis. The position increases linearly to about 80 millimeters at 20 percent, remains constant until 80 percent, then decreases linearly back to zero.

Navigate back to Figure 8..

## Figure 9. Long description

At the center, two red markers are placed on the scapular plane, connected by a horizontal orange arrow labeled interscapular distance. Above, C 7 is marked at the base of the neck. Moving outward and upward, T S and A A are labeled at the upper scapular region, with blue dots marking their positions. Below, T 7 is labeled at the lower thoracic spine, and A I is marked at the lower scapular angle. The schematic uses dashed lines to connect labels to anatomical points, and shaded areas indicate the scapulae.

Navigate back to Figure 9..

## Figure 12. Long description

At the top, the first sequence labeled N equals 5 shows four boxes in a row: Instruction, Self, Manual, Robot, separated by three vertical blue bars representing 10 minutes rest. Below, the second sequence labeled N equals 4 shows four boxes: Instruction, Self, Robot, Manual, also separated by three blue bars. From the Robot box, a dashed line branches downward to a sequence of three boxes: F V C, Stretching, F V C, with F V C and Stretching defined in the legend below. The legend at the bottom clarifies that F V C means F V C Measurement, the blue bar means 10 minutes rest, and Stretching means Stretching 10 times.

Navigate back to Figure 12..

## Table 1. Long description

From top to bottom, the table has two columns labeled List and Value. The first row under List is Max speed (no load) with Value 18 millimeters per second. The next row is Max force lifted (lowered) with Value 90 newtons. The following row is Back drive force with Value 200 newtons. The final row is Max static force with Value 500 newtons.

Navigate back to Table 1..
